# Family and Academic Stress and Their Impact on Students' Depression Level and Academic Performance

**DOI:** 10.3389/fpsyt.2022.869337

**Published:** 2022-06-16

**Authors:** Yuwei Deng, Jacob Cherian, Noor Un Nisa Khan, Kalpina Kumari, Muhammad Safdar Sial, Ubaldo Comite, Beata Gavurova, József Popp

**Affiliations:** ^1^School of Mechatronics Engineering, Daqing Normal University, Daqing, China; ^2^School of Marxism, Heilongjiang University, Harbin, China; ^3^College of Business, Abu Dhabi University, Abu Dhabi, United Arab Emirates; ^4^Faculty of Business Administration, Iqra University Karachi Pakistan, Karachi, Pakistan; ^5^Faculty of Department of Business Administration, Greenwich University Karachi, Karachi, Pakistan; ^6^Department of Management Sciences, COMSATS University Islamabad (CUI), Islamabad, Pakistan; ^7^Department of Business Sciences, University Giustino Fortunato, Benevento, Italy; ^8^Faculty of Mining, Ecology, Process Control and Geotechnologies, Technical University of Kosice, Kosice, Slovakia; ^9^Hungarian National Bank–Research Center, John von Neumann University, Kecskemét, Hungary; ^10^College of Business and Economics, University of Johannesburg, Johannesburg, South Africa

**Keywords:** academic stress, depression, university students, academic performance, academic learning, higher education, structural equation modeling (SEM)

## Abstract

Current research examines the impact of academic and familial stress on students' depression levels and the subsequent impact on their academic performance based on Lazarus' cognitive appraisal theory of stress. The non-probability convenience sampling technique has been used to collect data from undergraduate and postgraduate students using a modified questionnaire with a five-point Likert scale. This study used the SEM method to examine the link between stress, depression, and academic performance. It was confirmed that academic and family stress leads to depression among students, negatively affecting their academic performance and learning outcomes. This research provides valuable information to parents, educators, and other stakeholders concerned about their childrens' education and performance.

## Introduction

Higher education institutions (HEIs) are believed to be one of the strongest pillars in the growth of any nation (1). Being the principal stakeholder, the performance of HEIs mainly relies on the success of its students (2). To successfully compete in the prevailing dynamic industrial environment, students are not only supposed to develop their knowledge but are also expected to have imperative skills and abilities (3). In the current highly competitive academic environment, students' performance is largely affected by several factors, such as social media, academic quality, family and social bonding, etc. (4). Aafreen et al. (2) stated that students continuously experience pressure from different sources during academic life, which ultimately causes stress among students.

Stress is a common factor that largely diminishes individual morale (5). It develops when a person cannot handle their inner and outer feelings. When the stress becomes chronic or exceeds a certain level, it affects an individual's mental health and may lead to different psychological disorders, such as depression (6). Depression is a worldwide illness marked by feelings of sadness and the inability to feel happy or satisfied (7). Nowadays, it is a common disorder, increasing day by day. According to the World Health Organization (8, 9), depression was ranked third among the global burden of disease and predicted to take over first place by 2030.

Depression leads to decreased energy, difficulty thinking, concentrating, and making career decisions (6). Students are a pillar of the future in building an educated society. For them, academic achievement is a big goal of life and can severely be affected if the students fall prey to depression (10, 11). There can be several reasons for this: family issues, exposure to a new lifestyle in colleges and universities, poor academic grades, favoritism by teachers, etc. Never-ending stress or academic pressure of studies can also be a chief reason leading to depression in students (12). There is a high occurrence of depression in emerging countries, and low mental health literacy has been theorized as one of the key causes of escalating rates of mental illness (13).

Several researchers, such as (6, 14, 15) have studied stress and depression elements from a performance perspective and reported that stress and depression negatively affect the academic performance of students. However, Aafreen et al. (2) reported contradictory results and stated that stress sharpens the individual's mind and reflexes and enables workers to perform better in taxing situations. Ardalan (16) conducted a study in the United States (US). They reported that depression is a common issue among students in the US, and 20 percent of them may have a depressive disorder spanning 12 months or more. It affects students' mental and physical health and limits their social relationships and professional career.

However, the current literature provides mixed results on the relationship between stress and performance. Therefore, the current research investigates stress among students from family and academic perspectives using Lazaru's theory which describes stress as a relation between an individual and his environment and examines how it impacts students' depression level, leading to their academic performance. Most of the available studies on stress and depression are from industrial perspectives, and limited attention is paid to stress from family and institutional perspectives and examines its impact on students' depression level, leading to their academic performance, particularly in Pakistan, the place of the study. Besides, the present study follows a multivariate statistical technique, followed by structural equation modeling (SEM) to examine the relationship between stated variables which is also a study's uniqueness.

This paper is divided into five main sections. The current section provided introduction, theoretical perspective, and background of the study. In the second section, a theoretical framework, a detailed literature review and research hypotheses of the underlying relationships are being proposed. In the third and fourth section, methodology and analysis have been discussed. Finally, in the last section, the conclusion, limitations, implications, and recommendations for future research have been proposed.

## Theory and Literature

The idea of cognitive appraisal theory was presented in 1966 by psychologist Richard Lazarus in Psychological Stress and Coping Process. According to this theory, appraisal and coping are two concepts that are central to any psychological stress theory. Both are interrelated. According to the theory, stress is the disparity between stipulations placed on the individuals and their coping resources (17). Since its first introduction as a comprehensive theory (18), a few modifications have been experienced in theory later. The recent adaptation states that stress is not defined as a specific incitement or psychological, behavioral, or subjective response. Rather, stress is seen as a relation between an individual and his environment (19). Individuals appraise the environment as significant for their well-being and try to cope with the exceeding demands and challenges.

Cognitive appraisal is a model based on the idea that stress and other emotional processes depend on a person's expectancies regarding the significance and outcome of an event, encounter, or function. This explains why there are differences in intensity, duration, and quality of emotions elicited in people in response to the environment, which objectively, are equal for all (18). These appraisals may be influenced by various factors, including a person's goals, values, motivations, etc., and are divided into primary and secondary appraisals, specific patterns of which lead to different kinds of stress (20). On the other hand, coping is defined as the efforts made by a person to minimize, tolerate, or master the internal and external demands placed on them, a concept intimately related to cognitive appraisal and, therefore, to the stress-relevant person-environment transactions.

**Figure 1:**
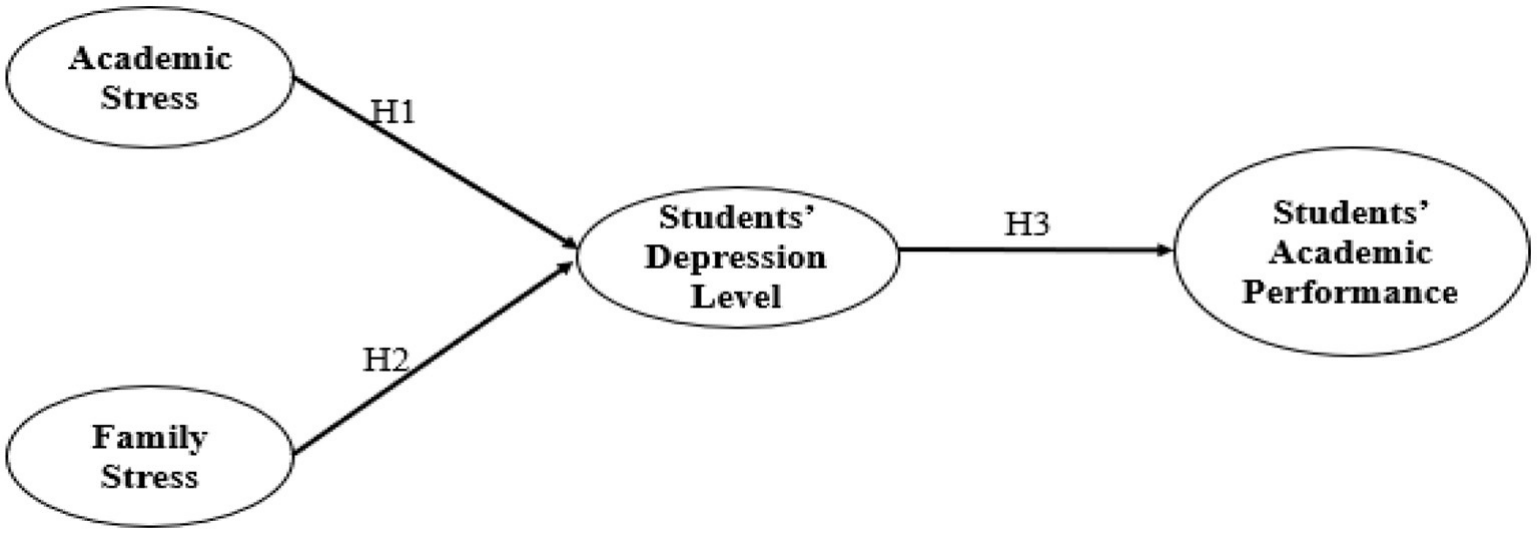
Conceptual framework.

Individuals experience different mental and physiological changes when encountering pressure, such as stress (21, 22). The feelings of stress can be either due to factors in the external environment or subjective emotions of individuals, which can even lead to psychological disorders such as anxiety and depression. Excess stress can cause health problems. A particularly negative impact has been seen in students due to the high level of stress they endure, affecting their learning outcomes. Various methods are used to tackle stress. One of the methods is trying to pinpoint the causes of stress, which leads us to different terms such as family stress and academic stress. The two factors, stress and depression, have greatly impacted the students' academic performances. This research follows the Lazarus theory based on stress to examine the variables. See the conceptual framework of the study in Figure 1.

### Academic Stress

Academic issues are thought to be the most prevalent source of stress for college students (23). For example, according to Yang et al. (24), students claimed that academic-related pressures such as ongoing study, writing papers, preparing for tests, and boring professors were the most important daily problems. Exams and test preparation, grade level competitiveness, and gaining a big quantity of knowledge in a short period of time all contribute to academic pressure. Perceived stress refers to a condition of physical or psychological arousal in reaction to stressors (25, 26). When college students face excessive or negative stress, they suffer physical and psychological consequences. Excessive stress can cause health difficulties such as fatigue, loss of appetite, headaches, and gastrointestinal issues. Academic stress has been linked to a variety of negative effects, including ill health, anxiety, depression, and poor academic performance. Travis et al. (27), in particular, discovered strong links between academic stress and psychological and physical health.

### Family Stress

Parental participation and learning effect how parents treat their children, as well as how they handle their children's habits and cognitive processes (28). This, in turn, shapes their children's performance and behaviors toward them. As a result, the parent-child relationship is dependent on the parents' attitudes, understanding, and perspectives. When parents have positive views, the relationship between them and their children will be considerably better than when they have negative attitudes. Parents respond to unpleasant emotions in a variety of ways, which can be classified as supportive or non-supportive (29). Parents' supportive reactions encourage children to explore their emotions by encouraging them to express them or by assisting them in understanding and coping with an emotion-eliciting scenario. Non-supportive behaviors, such as downplaying the kid's emotional experience, disciplining the child, or getting concerned by the child's display, transmit the child the message that expressing unpleasant emotions is inappropriate and unacceptable. Supportive parental reactions to unpleasant emotions in children have been linked to dimensions of emotional and social competence, such as emotion comprehension and friendship quality. Non-supportive or repressive parental reactions, on the other hand, have been connected to a child's stored negative affect and disordered behaviors during emotion-evoking events, probably due to an inability or unwillingness to communicate unpleasant sentiments (30, 31).

### Academic Stress and Students' Depression Levels

Generally, it is believed that mental health improves as we enter into adulthood, and depression disorder starts to decline between the age of 18 and 25. On the other hand, excessive depression rates are the highest pervasiveness during this evolution (15), and many university students in the particular screen above clinical cut-off scores for huge depression (14, 32). Afreen et al. (2) stated that 30% of high school students experience depression from different perspectives. This means a major chunk of fresh high school graduates are more likely to confront depression or are more vulnerable to encountering depression while enrolling in the university. As the students promote to a higher level of education, there are many factors while calculating the stress like, for example, the syllabus is tough to comprehend, assignments are quite challenging with unrealistic deadlines, and accommodation problems for the students who are shifted from other cities, etc. (33). Experiences related to university can also contribute while studying depression. The important thing to consider is depression symptoms vary from time to time throughout the academic years (34); subjective and objective experiences are directly connected to the depression disorder (6), stress inherent in the university situation likely donates to the difference in university students' depressing experiences.

Stress negatively impacts students' mental peace, and 42.3% of students of Canadian university respondents testified devastating levels of anxiety and stress (35, 36). Moreover, there were (58.1%) students who stated academic projects are too tough to handle for them. In Germany, Bulgaria, and Poland, a huge sample of respondents consider assignments a burden on their lives that cannot stand compared to relationships or any other concern in life (14).

In several countries, university students were studied concerning stress, and results show that depression disorder and apparent anxiety are correlated to educational needs and demands (37). In their cross-sectional study conducted on a sample of 900 Canadian students, Lörz et al. (38) concluded that strain confronted due to academic workload relatively has high bleak symptoms even after controlling 13 different risk affecting factors for depression (e.g., demographic features, abusive past, intellectual way, and personality, currently experienced stressful trials in life, societal support). Few have exhibited that students who are tired of educational workload or the students who name them traumatic tend to have more depressing disorders (15).

These relations can be described by examining the stress and coping behaviors that highlight the role of positive judgments in the stress times (39), containing the Pancer and colleagues' university modification framework (40, 41). The evaluation concept includes examining the circumstances against the available resources, for instance, the effectiveness of coping behavior and societal support. As per these frameworks, if demand is considered unapproachable and resources are lacking, confronted stress and interrelated adverse effects will be high, conceivably giving birth to difficulties in an adjustment like mental instability. Stress triggering situations and the resources in the educational area led to excessive workload, abilities, and study and enhanced time managing skills.

Sketching the overall evaluation frameworks, Pancer et al. (40) established their framework to exhibit the constructive and damaging adjustment results for the university students dealing with the academic challenges. They stated that while students enroll in the university, they evaluate all the stress-related factors that students confront. They consider them manageable as long as they have sufficient resources. On the other hand, if the available resources do not match the stress factors, it will surely result in a negative relationship, which will lead students to experience depression for sure. Based on the given arguments, the researcher formulates the following hypothesis:

H1: Increased academic stress results in increased depression levels in students.

### Family Stress and Students' Depression Levels

According to Topuzoglu et al. (42), 3% to 16.9% of individuals are affected by depression worldwide. There are fewer chances for general people to confront depression than university students (43, 44). In Mirza et al.'s (45) study, 1/3 of students encounter stress and depression (a subjective mean occurrence of 30.6%) of all participant students, which suggests students have a 9% higher rate of experiencing depression than general people. Depression can destroy life; it greatly impacts living a balanced life. It can impact students' personal and social relationships, educational efficiency, quality of life, affecting their social and family relationships, academic productivity, and bodily operations (46, 47). This declines their abilities, and they get demotivated to learn new things, resulting in unsatisfactory performances, and it can even result in university dropouts (48). Depression is a continuous substantial risk aspect for committing suicide for university students (49); thus, it is obliged to discover the factors that can give rise to students' depression.

Seventy-five percentage of students in China of an intermediate school are lucky enough to enroll in higher education. The more students pursue higher education, the more they upsurge for depression (in 2002, the depression rate was 5 to 10%, 2011 it rises 24 to 38%) (5). Generally, University students' age range is late teens to early twenties, i.e., 18–23 years. Abbas (50) named the era of university students as “post-adolescence. Risk factors for teenage depression have several and complicated problems of individual characteristics and family and educational life (51). Amongst the huge depression factors, relationship building with family demands a major chunk of attention and time since factors like parenting and family building play an important role in children's development (52, 53). Halonen et al. (54) concluded that factors like family binding play a major role in development, preservation, and driving adolescent depression. Generally speaking, depressed teenagers tend to have a weaker family relationship with their parents than non-depressed teenagers.

There are two types of family risk factors, soft and hard. Hard factors are encountered in families with a weak family building structure, parents are little to no educated at all, and of course, the family status (economically). Several studies have proved that students of hard risk factors are more likely to encounter depression. Firstly, students from broken families have low confidence in every aspect of life, and they are weak at handling emotional breakdowns compared to students from complete and happy families (55–57). Secondly, the university students born in educated families, especially mothers (at least a college degree or higher degree), are less likely to confront depression than the university students born in families with little to no educated families. Secondly, children born with educated mothers or mothers who at least have a college degree tend to be less depressive than the children of less-educated mothers (58). However, Parker et al. and Mahmood et al. (59, 60) stated a strong relationship between depression and mothers with low literacy levels.

On the other hand, Chang et al. (46) couldn't prove the authentication of this relationship in university students. Thirdly, university students who belong to lower class families tend to have more unstable mental states and are more likely to witness depression than middle or upper-class families (61). Jadoon et al. and Abbas et al. (62, 63) said that there is no link between depression and economic status. Their irrelevance can be because medical students often come from educated and wealthy families and know their jobs are guaranteed as soon as they graduate. Therefore, the relationship between the hard family environment and depression can be known by targeting a huge audience, and there are several factors to consider while gauging this relationship.

The soft family environment is divided into clear factors (parenting style example, family guidelines, rules, the parent with academic knowledge, etc.) and implied factors (family norm, parent-child relationship, communication within the family, etc.). The soft factor is the key factor within the family that cannot be neglected while studying the teenagers' mental state or depression. Families make microsystems within the families, and families are the reason to build and maintain dysfunctional behavior by multiple functional procedures (64). Amongst the soft family environmental factors, consistency and struggles can be helpful while forecasting the mental health of teenagers. The youth of broken families, family conflict, weak family relationships, and marital issues, especially unhappy married life, are major factors for youth depression (65). Ruchkin et al. (66) stated that African Americans usually have weak family bonding, and their teenagers suffer from depression even when controlling for source bias. Whereas, few researchers have stated, family unity is the most serious factor while foreseeing teenagers' depression. Eaton noted that extreme broken family expressions might hurt emotionality and emotional regulation (67, 68).

Social circle is also considered while studying depression in teenagers (69–71). The traditional Pakistani culture emphasizes collectivism and peace and focuses on blood relations and sensitive sentiments. Adolescents with this type of culture opt to get inspired by family, but students who live in hostels or share the room with other students lose this family inspiration. This transformation can be a big risk to encounter depression (72). Furthermore, in Pakistan securing employment is a big concern for university students. If they want a good job in the future, they have to score good grades and maintain GPA from the beginning. They have to face different challenges all at once, like aggressive educational competition, relationships with peers and family, and of course the biggest employment stress all alone. The only source for coping with these pressures is the family that can be helpful for fundings. If the students do not get ample support the chances are of extreme depression. The following hypothesis is suggested:

H2: Increased family stress level results in increased depression levels in students.

### Students' Depression Levels and Students' Academic Performance

University students denote many people experiencing a crucial conversion from teenagers to adulthood: a time that is generally considered the most traumatic time in one's (73). This then gets accumulated with other challenges like changes in social circle and exams tension, which possibly puts students' mental health at stake. It has been concluded that one-third of students experience moderate to severe depression in their entire student life (74). This is the rate that can be increased compared to the general people (75, 76). Students with limited social-class resources tend to be more helpless. Additionally, depressed students in attainable-focused environments (for instance, higher academic institutes) are likely to score lower grades with a sense of failure and more insufficient self-assurance because they consider themselves failures, find the world unfair, and have future uncertainties. Furthermore, students with low self-esteem are rigid to take on challenging assignments and projects, hence they are damaging their educational career (77).

Depression can be defined as a blend of physical, mental, bodily processes, and benightedness which can make themselves obvious by symptoms like, for example, poor sleep schedule, lack of concentration, ill thoughts, and state of remorse (78, 79). But, even after such a huge number of depressions in students and the poor academic system, research has not explored the effect of depression on educational performance. A study has shown that the relationship between emotional stability and academic performance in university students and financial status directly results in poor exam performance. As the study further concluded, it was verified depression is an independent factor (80). Likewise, students suffering from depression score poor grades, but this relationship vanished if their depression got treated. Apart from confidence breaking, depression is a big failure for their academic life. Students with depression symptoms bunk more classes, assessments, and assignments. They drop courses if they find them challenging than non-depressed peers, and they are more likely to drop out of university completely (81). Students suffering from depression can become ruthless, ultimately affecting their educational performance and making them moody (82).

However, it has been stated that the association between anxiety and educational performance is even worse and ambiguous. At the same time, some comprehensive research has noted that the greater the anxiousness, the greater the student's performance. On the other hand, few types of research have shown results where there is no apparent relationship between anxiety and poorer academic grades (83). Ironically, few studies have proposed that a higher anxiety level may improve academic performance (84, 85). Current research by Khan et al. (86) on the undergraduate medical students stated that even though the high occurrence of huge depression between the students, the students GPA is unharmed. Therefore, based on given differences in various research findings, this research is supposed to find a more specific and clear answer to the shared relationship between students' depression levels and academic performance. Based on the given arguments, the researcher formulates the following hypothesis:

H3: Students' depression level has a significant negative effect on their academic performance.

## Methodology

### Target Population and Sampling Procedure

The target audience of this study contains all male and female students studying in the public, private, or semi-government higher education institutions located in Rawalpindi/Islamabad. The researchers collected data from undergraduate and postgraduate students from the management sciences, engineering, and computer science departments. The sampling technique which has been used is the non-probability sampling technique. A questionnaire was given to the students, and they were requested to fill it and give their opinion independently. The questionnaire is based on five points Likert scale.

However, stress and depression are the most common issue among the students, which affects their learning outcomes adversely. A non-probability sampling technique gathered the data from February 2020 to May 2020. The total questionnaires distributed among students were 220, and 186 responses were useful. Of which 119 respondents were females, 66 males, and 1 preferred not to disclose. See Table 1 for detailed demographic information of respondents.

**Table 1:** Respondent's demographic profile.

**Particulars**	**Description**	**Values**	**Percentage**
Total received responses	Public	36	19.35%
	Private	117	62.90%
	Semi-government	33	17.74%
Gender	Male	66	35.48%
	Female	119	63.97%
	Prefer not to disclose	1	0.54%
Age	Less than 20	29	15.59%
	21–30	146	78.49%
	31–40	11	5.91%
Qualification	Undergraduates	116	62.36%
	Postgraduates	70	37.63%
Degree Program	Management sciences	68	36.55%
	Engineering	8	4.30%
	Computer science	21	11.29%
	Others	89	47.84%
Semester	1^st^ year	23	12.36%
	2^nd^ year	40	21.50%
	3^rd^ year	37	19.89%
	4^th^ year	48	25.80%
	More than 4^th^ year	38	20.43%

### Measurement Scales

We have divided this instrument into two portions. In the first section, there is demographic information of respondents. The second section includes 14 items based on family stress, academic stress, students' depression levels, and students' academic performance. Academic and family stress were measured by 3 item scale for each construct, and students' depression level and academic performance were measured by 4 item scale for each separate construct. The five-point Likert scale is used to measure the items, in which one signifies strongly disagree (S.D), second signifies disagree (D.A), third signifies neither agree nor disagree (N), fourth signifies agree (A.G), and the fifth signifies strongly agree (S.A). The questionnaire has been taken from Gold Berg (87), which is modified and used in the given questionnaire.

## Data Analysis and Results

The researchers used the SEM technique to determine the correlation between stress, depression, and academic performance. According to Prajogo and Cooper (88), it can remove biased effects triggered by the measurement faults and shape a hierarchy of latent constructs. SPSS v.23 and AMOS v.23 have been used to analyze the collected data. Kaiser-Meyer-Olkin test is used to test the competence of the sample. The value obtained is 0.868, which fulfills the Kaiser et al. (89), a minimum requirement of 0.6. The multicollinearity factor was analyzed through the variance inflation factor (VIF). It shows the value of 3.648 and meets the requirement of Hair et al. (90), which is < 4. It also indicates the absence of multicollinearity. According to Schwarz et al. (91), common method bias (CMB) is quite complex in quantitative studies. Harman's test of a single factor has been used to analyze CMB. The result obtained for the single factor is 38.63%. As stated by Podsakoff et al. (92), if any of the factors gives value < 50% of the total variance, it is adequate and does not influence the CMB. Therefore, we can say that there is no issue with CMB. Considering the above results are adequate among the measurement and structural model, we ensure that the data is valued enough to analyze the relation.

### Assessment of the Measurement and Structural Model

The association between the manifest factors and their elements is examined by measuring model and verified by the Confirmatory Factor Analysis (CFA). CFA guarantees legitimacy and the unidimensional of the measurement model (93). Peterson (94) stated that the least required, i.e., 0.8 for the measurement model, fully complies with its Cronbach's alpha value, i.e., 0.802. Therefore, it can confidently be deduced that this measurement model holds satisfactory reliability. As for the psychological legitimacy can be analyzed through factor loading, where the ideal loading is above 0.6 for already established items (95). Also, according to the recommendation of Molina et al. (96), the minimum value of the average variance extracted (AVE) for all results is supposed to be >0.5. Table 2 gives detail of the variables and their quantity of things, factor loading, merged consistency, and AVE values.

**Table 2:** Instrument reliability and validity.

**Variable**	**No. of items**	**Factor loading**	**Composite^**a**^ reliability**	**AVE^**b**^**
Academic stress	3	0.818–0.941	0.863	0.698
Family stress	3	0.852–0.897	0.778	0.721
Student's depression level	4	0.776–0.921	0.897	0.685
Student's academic performance	4	0.779–0.918	0.914	0.693

a *Composite reliability should be > 0.7 (96).*

b *The average variance extracted (AVE) value should also be > 0.5 (96)*.

A discriminant validity test was performed to ensure the empirical difference of all constructs. For this, it was proposed by Fornell and Larcker (97) that the variance of the results is supposed to be greater than other constructs. The second indicator of discriminant validity is that the square root values of AVE have a greater correlation between the two indicators. Hair et al. (90) suggested that the correlation between the pair of predictor variables should not be higher than 0.9. Table 3 shows that discriminant validity recommended by Hair et al. (90) and Fornell and Larcker (97) was proved clearly that both conditions are fulfilled and indicates that the constructs have adequate discriminant validity.

**Table 3:** Discriminant validity analysis.

**Variable**	**Acd. Strs**	**Fam. Strs**	**Std.Dep. Lev**	**Std. Acd. Perf**
Acd. Strs	0.835			
Fam. Strs	0.543	0.849		
Std. Dep. Lev	0.622	0.583	0.827	
Std. Acd. Perf	0.623	0.629	0.579	0.832

*Acd. Strs, Academic Stress; Fam. Strs, Family Stress; Std. Dep. Lev, Student's Depression Level; Std. Acd. Perf, Student's Academic Performance*.

Kaynak (98) described seven indicators that ensure that the measurement model fits correctly. These indicators include standardized root mean squared residual (SRMR), root means a square error of approximation (RMSEA), comparative fit index (CFI), normative fit index (NFI), adjusted goodness of fit index (AGFI), the goodness of fit index (GFI) and chi-square to a degree of freedom (x^2^/DF). Tucker-Lewis's index (TLI) is also included to ensure the measurement and structural model's fitness. In the measurement model, the obtained result shows that the value of x^2^/DF is 1.898, which should be lower than 2 suggested by Byrne (99), and this value also meets the requirement of Bagozzi and Yi (100), i.e., <3. The RMSEA has the value 0.049, which fully meets the requirement of 0.08, as stated by Browne and Cudeck (101). Furthermore, the SRMR acquired value is 0.0596, which assemble with the required need of < 0.1 by Hu and Bentler (102). Moreover, according to Bentler and Bonett (103), McDonald and Marsh (104), and Bagozzi and Yi (100), the ideal value is 0.9, and the values obtained from NFI, GFI, AGFI, CFI, and TLI are above the ideal value.

Afterward, the structural model was analyzed and achieved the findings, which give the value of x^2^/DF 1.986. According to Browne and Cudeck (101), the RMSEA value should not be greater than 0.08, and the obtained value of RMSEA is 0.052, which meets the requirement perfectly. The minimum requirement of Hu and Bentler (102) should be <0.1, for the structural model fully complies with the SRMR value 0.0616. According to a recommendation of McDonald and Marsh (104) and Bagozzi and Yi (100), the ideal value must be up to 0.9, and Table 4 also shows that the values of NFI, GFI, AGFI, CFI, and TLI, which are above than the ideal value and meets the requirement. The above results show that both the measurement and structural models are ideally satisfied with the requirements and the collected data fits correctly.

**Table 4:** Analysis of measurement and structural model.

**The goodness of fit measures**	**CMIN/DF**	**NFI**	**GFI**	**AGFI**	**CFI**	**TLI**	**RMSEA**	**SRMR**
Recommended value	≤ 3^a^	≥0.9^b^	≥0.9^b^	≥0.9^b^	≥0.9^b^	≥0.9^b^	≤0.08^c^	≤0.08^*d*^
Measurement model	1.898	0.9	0.91	0.914	0.91	0.91	0.049	0.0596
Structural model	1.986	0.91	0.91	0.918	0.92	0.92	0.052	0.0616

a *(100).*

b *(103, 104).*

c *(101).*

d *(102)*.

### Testing of Hypotheses

**Figure 2:**
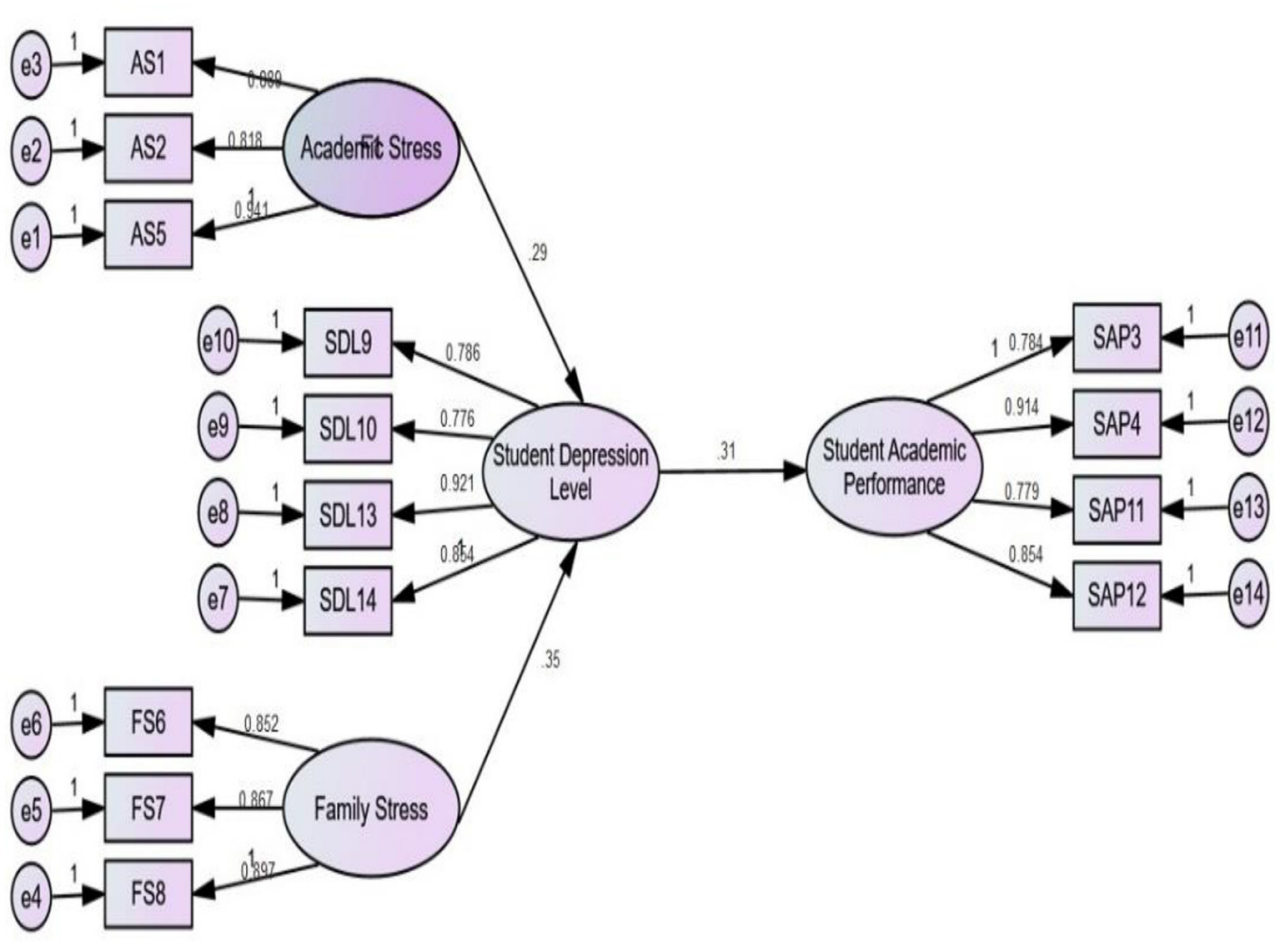
Structural model.

The SEM technique is used to examine the hypotheses. Each structural parameter goes along with the hypothesis. The academic stress (Acd. Strs) with the value β = 0.293 while the *p*-value is 0.003. These outcomes show a significant positive relationship between academic stress (Acd. Strs) and students' depression levels (Std. Dep. Lev). With the β = 0.358 and *p* = 0.001 values, the data analysis discloses that the family stress (Fam. Strs) has a significant positive effect on the students' depression level (Std. Dep. Lev). However, the student's depression level (Std. Dep. Lev) also has a significant negative effect on their academic performance (Std. Acd. Perf) with the values of β = −0.319 and *p* = 0.001. Therefore, the results supported the following hypotheses H_1_, H_2_, and H_3_. The sub-hypotheses analysis shows that the results are statistically significant and accepted. In Table 5, the details of the sub-hypotheses and the principals are explained precisely. Please see Table 6 to review items with their mean and standard deviation values. Moreover, Figure 2 represents the structural model.

**Table 5:** Examining the hypotheses.

**Hypothesis**	**Constructs**	**Estimate**	**Critical ratio**	***p*-value**	**Decision**
H_1_	Acd. Strs → Std. Dev. Lev	0.201	2.021	0.039*	Accepted
H_2_	Fam. Strs → Std. Dep. Lev	0.358	3.997	0.001*	Accepted
H_3_	Std. Dep. Lev → Std. Acd. Perf	−0.319	−3.402	0.001*	Accepted

* *Acd. Strs, Academic Stress; Fam. Strs, Family Stress; Std. Dep. Lev, Students' Depression Level; Std. Acd. Perf, Student's Academic Performance.*

* The value of p should be p* ≤ 0.05.

**Table 6:** Description of items, mean, and standard deviation.

**Items**	**Mean**	**Standard deviation**
Mental health has a valuable impact on students' academic learning.	3.26	1.752
Academic pressure leads to stress in students' life.	3.25	1.530
I have difficulty in understanding basic concepts.	2.95	1.272
I have to revise the things again and again to develop an understanding.	3.14	1.352
I have lost interest in academic aspects that used to be important for me.	2.83	1.351
Family issues leads to stress in students' life.	3.37	1.504
Because of family issues I cannot concentrate on my studies.	3.19	1.468
I am not able to sleep properly because of family issues.	3.02	1.424
Depression negatively affects a student's motivation to learn.	3.37	1.405
Unfair treatment by teachers causes academic depression in students.	3.12	1.620
Depression has negatively affected my learning capabilities.	2.99	1.280
Depression has negatively affected my academic grades.	3.19	1.201
Sometimes I don't see value in my life. I feel depressed in the class.	2.96 2.91	1.398 1.310

## Discussion and Conclusion

These findings add to our knowledge of how teenage depression is predicted by academic and familial stress, leading to poor academic performance, and they have practical implications for preventative and intervention programs to safeguard adolescents' mental health in the school context. The outcomes imply that extended academic stress positively impacts students' depression levels with a β of 0.293 and a *p*-value sof 0.003. However, according to Wang et al. (5), a higher level of academic stress is linked to a larger level of school burnout, which leads to a higher degree of depression. Satinsky et al. (105) also claimed that university officials and mental health specialists have expressed worry about depression and anxiety among Ph.D. students, and that his research indicated that depression and anxiety are quite common among Ph.D. students. Deb et al. (106) found the same results and concluded that depression, anxiety, behavioral difficulties, irritability, and other issues are common among students who are under a lot of academic stress. Similarly, Kokou-Kpolou et al. (107) revealed that depressive symptoms are common among university students in France. They also demonstrate that socioeconomic and demographic characteristics have a role.

However, Wang et al. (5) asserted that a higher level of academic stress is associated with a higher level of school burnout, which in return, leads to a higher level of depression. Furthermore, Satinsky et al. (105) also reported that university administrators and mental health clinicians have raised concerns about depression and anxiety and concluded in his research that depression and anxiety are highly prevalent among Ph.D. students. Deb et al. (106) also reported the same results and concluded that Depression, anxiety, behavioral problems, irritability, etc. are few of the many problems reported in students with high academic stress. Similary, Kokou-Kpolou et al. (107) confirmed that university students in France have a high prevalence of depressive symptoms. They also confirm that socio-demographic factors and perceived stress play a predictive role in depressive symptoms among university students. As a result, academic stress has spread across all countries, civilizations, and ethnic groups. Academic stress continues to be a serious problem impacting a student's mental health and well-being, according to the findings of this study.

With the β= 0.358 and *p* = 0.001 values, the data analysis discloses that the family stress (Fam. Strs) has a significant positive effect on the students' depression level (Std. Dep. Lev). Aleksic (108) observed similar findings and concluded that many and complicated concerns of personal traits, as well as both home and school contexts, are risk factors for teenage depression. Similarly, Wang et al. (109) indicated that, among the possible risk factors for depression, family relationships need special consideration since elements like parenting styles and family dynamics influence how children grow. Family variables influence the onset, maintenance, and course of juvenile depression, according to another study (110). Depressed adolescents are more likely than normal teenagers to have bad family and parent–child connections.

Conversely, students' depression level has a significantly negative impact on their academic performance with β and *p*-values of −0.319 and 0.001. According (111), anxiety and melancholy have a negative influence on a student's academic performance. Adolescents and young adults suffer from depression, which is a common and dangerous mental illness. It's linked to an increase in family issues, school failure, especially among teenagers, suicide, drug addiction, and absenteeism. While the transition to adulthood is a high-risk period for depression in general (5), young people starting college may face extra social and intellectual challenges that increase their risk of melancholy, anxiety, and stress (112). Students' high rates of depression, anxiety, and stress have serious consequences. Not only may psychological morbidity have a negative impact on a student's academic performance and quality of life, but it may also disturb family and institutional life (107). Therefore, long-term untreated depression, anxiety, or stress can have a negative influence on people's ability to operate and produce, posing a public health risk (113).

### Theoretical Implications

The current study makes various contributions to the existing literature on servant leadership. Firstly, it enriches the limited literature on the role of family and academic stress and their impact on students' depression levels. Although, a few studies have investigated stress and depression and its impact on Students' academic performance (14, 114), however, their background i.e., family and institutions are largely ignored.

Secondly, it explains how the depression level impacts students' academic learning, specifically in the Asian developing countries region. Though a substantial body of empirical research has been produced in the last decade on the relationship between students' depression levels and its impact on their academic achievements, however, the studies conducted in the Pakistani context are scarce (111, 115). Thus, this study adds further evidence to prior studies conducted in different cultural contexts and validates the assumption that family and academic stress are key sources depression and anxiety among students which can lead toward their low academic grades and their overall performance.

This argument is in line with our proposed theory in the current research i.e., cognitive appraisal theory which was presented in 1966 by psychologist Richard Lazarus. Lazarus's theory is called the appraisal theory of stress, or the transactional theory of stress because the way a person appraises the situation affects how they feel about it and consequently it's going to affect his overall quality of life. In line with the theory, it suggests that events are not good or bad, but the way we think about them is positive or negative, and therefore has an impact on our stress levels.

### Practical Implications

According to the findings of this study, high levels of depressive symptoms among college students should be brought to the attention of relevant departments. To prevent college student depression, relevant departments should improve the study and life environment for students, try to reduce the generation of negative life events, provide adequate social support for students, and improve their cognitive and coping capacities to improve their mental qualities.

Stress and depression, on the other hand, may be managed with good therapy, teacher direction, and family support. The outcomes of this study provide an opportunity for academic institutions to address students' psychological well-being and requirements. Emotional well-being support services for students at Pakistan's higher education institutions are lacking in many of these institutions, which place a low priority on the psychological requirements of these students. As a result, initiatives that consistently monitor and enhance kids' mental health are critical. Furthermore, stress-reduction treatments such as biofeedback, yoga, life-skills training, mindfulness meditation, and psychotherapy have been demonstrated to be useful among students. Professionals in the sector would be able to adapt interventions for pupils by understanding the sources from many spheres.

Counseling clinics should be established at colleges to teach students about stress and sadness. Counselors should instill in pupils the importance of positive conduct and decision-making. The administration of the school should work to create a good and safe atmosphere. Furthermore, teachers should assume responsibility for assisting and guiding sad pupils, since this will aid in their learning and performance. Support from family members might also help you get through difficult times.

Furthermore, these findings support the importance of the home environment as a source of depression risk factors among university students, implying that family-based treatments and improvements are critical in reducing depression among university students.

### Limitations and Future Research Implications

The current study has a few limitations. The researcher gathered data from the higher education level of university students studying in Islamabad and Rawalpindi institutions. In the future, researchers are required to widen their region and gather information from other cities of Pakistan, for instance, Lahore, Karachi, etc. Another weakness of the study is that it is cross-sectional in nature. We need to do longitudinal research in the future to authoritatively assert the cause-and-effect link between academic and familial stress and their effects on students' academic performance since cross-sectional studies cannot establish significant cause and effect relationships. Finally, the study's relatively small sample size is a significant weakness. Due to time and budget constraints, it appears that the capacity to perform in-depth research of all firms in Pakistan's pharmaceutical business has been limited. Even though the findings are substantial and meaningful, the small sample size is predicted to limit generalizability and statistical power. This problem can be properly solved by increasing the size of the sample by the researchers, in future researches.

## Data Availability Statement

The raw data supporting the conclusions of this article will be made available by the authors, without undue reservation.

## Ethics Statement

Ethical review and approval was not required for the study on human participants in accordance with the local legislation and institutional requirements. Written informed consent for participation was not required for this study in accordance with the national legislation and the institutional requirements.

## Author Contributions

All authors contributed to conceptualization, formal analysis, investigation, methodology, writing and editing of the original draft, and read and agreed to the published version of the manuscript.

## Funding

This work was funded by the 2020 Heilongjiang Province Philosophy and Social Science Research Planning Project on Civic and Political Science in Universities (Grant No. 20SZB01). This work is supported by the Scientific Grant Agency of the Ministry of Education, Science, Research, and Sport of the Slovak Republic and the Slovak Academy Sciences as part of the research project VEGA 1/0797/20: Quantification of Environmental Burden Impacts of the Slovak Regions on Health, Social and Economic System of the Slovak Republic.

## Conflict of Interest

The authors declare that the research was conducted in the absence of any commercial or financial relationships that could be construed as a potential conflict of interest.

## Publisher's Note

All claims expressed in this article are solely those of the authors and do not necessarily represent those of their affiliated organizations, or those of the publisher, the editors and the reviewers. Any product that may be evaluated in this article, or claim that may be made by its manufacturer, is not guaranteed or endorsed by the publisher.
